# Patient Beliefs on Antibiotic Prescribing in Primary Care: A Cross-Sectional Survey in Saudi Arabia

**DOI:** 10.7759/cureus.38254

**Published:** 2023-04-28

**Authors:** Mohammed S Fallatah, Abdulaziz A Alzahrani, Ghassan S Alghamdi, Mohannad M Sadagah, Turki M Alkharji

**Affiliations:** 1 General Practice, King Abdulaziz University, Jeddah, SAU; 2 General Practice, Jeddah University, Jeddah, SAU; 3 Family Medicine, Al Thaghr Hospital, Jeddah, SAU

**Keywords:** primary care, patient education, respiratory tract infections, cross-sectional survey, antibiotic resistance, antibiotic knowledge, patient expectations

## Abstract

Background

Antibiotic overuse is a critical global health issue, and patient attitudes and expectations play a significant role in the inappropriate use of antibiotics. Limited research has been conducted on patient knowledge, attitudes, and perceptions of antibiotic use in Saudi Arabia. This survey aimed to assess patients' knowledge and attitudes related to antibiotic use in Jeddah, Saudi Arabia.

Methods

A cross-sectional survey using a convenience sampling method was conducted in Saudi Arabia. An online self-administered questionnaire was used to collect demographic data, antibiotic knowledge, and attitudes.

Results

The study included 400 patients, with a mean age of 39 years and an equal gender distribution (54% female). Most participants (75%) had not used antibiotics in the past year. Patients demonstrated moderate knowledge about antibiotics, with 81% recognizing that antibiotics can cause side effects and 69% knowing that overuse can lead to resistance. However, only 44% knew that antibiotics are not effective for all infections, and only half (50%) knew that antibiotics work against bacteria, not viruses. Patients held mixed attitudes toward antibiotic prescribing, with 25% believing it was essential to take antibiotics for every infection and 44% believing healthcare providers should prescribe antibiotics for respiratory tract infections. Logistic regression analyses showed that patient expectations for antibiotic prescribing were strongly associated with inappropriate antibiotic use. In contrast, patient satisfaction with antibiotic prescribing was negatively associated with inappropriate antibiotic use. Lower health literacy levels were also associated with inappropriate antibiotic use.

Conclusion

The study underscores the need for interventions that promote patient education and communication to ensure appropriate antibiotic use in primary care. Patient attitudes and beliefs, such as their expectations for antibiotic prescribing and health literacy levels, were identified as significant predictors of inappropriate antibiotic use.

## Introduction

Antimicrobial resistance poses a substantial hazard to public health, impacting all countries. It is estimated that it will account for 10 million fatalities annually by 2050 [1]. One of the major drivers of antibiotic resistance is the inappropriate use of antibiotics in human and animal health [1,2]. Inappropriate use includes overuse, misuse, and unnecessary use of antibiotics [2,3].

In primary care settings, antibiotics are frequently prescribed for respiratory tract infections, despite most of these infections being viral and not requiring antibiotics [2]. Patient expectations and beliefs about antibiotics have been identified as significant contributors to antibiotic overuse in primary care [2,3]. Studies have shown that patients often expect to receive antibiotics for respiratory tract infections, and healthcare providers may prescribe antibiotics even if they are not necessary to meet patient expectations [4-6].

Additionally, patient knowledge about antibiotics and their consequences can influence prescribing practices [3-5]. Poor patient understanding of the risks of antibiotic overuse and the development of antibiotic resistance may lead to increased antibiotic prescribing by healthcare providers [5-7]. In Saudi Arabia, there is a high prevalence of antibiotic use, with antibiotics being readily available without a prescription [3]. This may lead to increased antibiotic resistance and has become a major public health concern [3,8].

To address this growing concern, it is essential to understand patient perceptions and attitudes toward antibiotic use in primary care settings. A cross-sectional study conducted in Saudi Arabia aims to assess patient knowledge, attitudes, and perceptions of antibiotic use in primary care. The study will involve surveying patients attending primary care clinics and will focus on identifying factors that influence patient expectations for antibiotics, as well as patient knowledge about antibiotic resistance and appropriate antibiotic use.

## Materials and methods

Study design and setting

This cross-sectional survey aimed to assess patients' knowledge and attitudes related to antibiotic use in Jeddah, Saudi Arabia. The study was conducted in accordance with the principles outlined in the Declaration of Helsinki.

Study participants

Adult patients who had visited any primary care clinic in Jeddah within the past six months were eligible to participate in this study. Participants were recruited using a convenience sampling method through an online survey platform, which was distributed through social media platforms, online groups, and forums related to healthcare and medicine. Participants were informed that participation was voluntary, and informed consent was obtained from all participants before they started the survey.

Survey instrument

For this study, an online self-administered questionnaire was developed by the researchers to collect data on patients' antibiotic knowledge, attitudes, and practices. The questionnaire was divided into three sections, including demographic information such as age, gender, educational level, income, and occupation; antibiotic knowledge, which included questions about antibiotics and their usage, benefits, risks, and appropriate use; and antibiotic attitudes and practices, which included questions about patients' attitudes toward antibiotic use, perceptions about the effectiveness of antibiotics, willingness to follow physician advice on antibiotic use, and previous experiences with antibiotic use. The knowledge subsection included questions about what antibiotics are used for, whether antibiotics are effective for all infections, whether antibiotics can cause side effects, and whether participants knew what antibiotic resistance is and that overuse of antibiotics can lead to resistance. The attitudes subsection included questions about the importance of taking antibiotics for every infection, whether healthcare providers should prescribe antibiotics for respiratory tract infections, whether participants would be dissatisfied if their healthcare provider did not prescribe antibiotics for a respiratory tract infection, and whether participants expected their healthcare provider to prescribe antibiotics if they ask for them. The questionnaire was developed based on a literature review and was reviewed by two experts in the field of family medicine for face validity. A pilot test was conducted on a sample of 20 patients to assess its reliability and validity.

Data collection

Data was collected using the online survey platform (Google Forms). The survey link was distributed through various online platforms related to healthcare and medicine. Participants were requested to share the survey link with others who may be interested in participating. However, only responses meeting the criteria were considered in the data analysis. The survey was open for two months, from January 1, 2023, to February 28, 2023. The responses were anonymous, and the participants were assured of confidentiality.

Data analysis

Descriptive statistics were used to summarize the demographic characteristics of the sample and to describe patient knowledge and attitudes toward antibiotic use. Logistic regression analysis was used to examine the factors associated with inappropriate antibiotic use, including patient expectations and satisfaction with prescribing practices, as well as health literacy levels. The data analysis was conducted using statistical software, and a p-value of less than 0.05 was considered statistically significant. The data analysis was done using SPSS Statistics version 26 (IBM Corp. Released 2019. IBM SPSS Statistics for Windows, Version 26.0. Armonk, NY: IBM Corp).

## Results

Demographics of the study population

Table 1 summarizes the characteristics of the 400 patients who completed the survey. The mean age of the participants was 39 years, with a standard deviation of 12.5 years. The gender distribution was roughly equal, with 216 (54%) participants identifying as female. The majority of participants had not used antibiotics in the past year, with 301 (75%) participants reporting no antibiotic use.

**Table 1:** Demographic characteristics

Demographic Characteristics		Frequency	Percent
Age Group	18-29	60	15%
	30-39	124	31%
	40-49	100	25%
	50-59	76	19%
	60 and above	40	10%
Gender	Male	184	46%
	Female	216	54%
Education Level	High School or less	100	25%
	Diploma Degree	152	38%
	Bachelor's Degree	100	25%
	Master’s Degree or PhD	48	12%

Antibiotic knowledge of study participants

**Figure 1:**
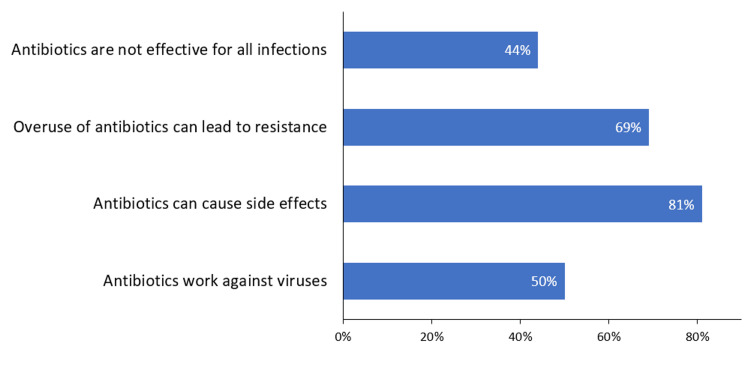
Bar chart displaying the frequency of responses from participants regarding statements related to antibiotic knowledge

Overall, patients had moderate knowledge about antibiotics. Most participants correctly identified that antibiotics can cause side effects (n=324, 81%) and that overuse of antibiotics can lead to resistance (n=276, 69%). However, fewer patients correctly identified that antibiotics are not effective for all infections (n=176, 44%). Additionally, only half (n=201, 50%) of the participants recognized that antibiotics work against bacteria, not viruses (Figure 1).

Attitudes toward antibiotic use

**Figure 2:**
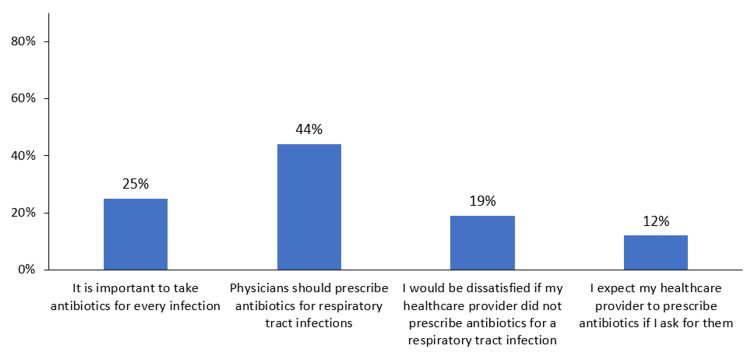
Column chart depicting the distribution of responses from participants regarding statements related to antibiotic attitude

Patients expressed a mix of attitudes toward antibiotic prescribing. A significant proportion of patients (n=99, 25%) believed that it was important to take antibiotics for every infection, and under half of participants (n=176, 44%) believed that healthcare providers should prescribe antibiotics for respiratory tract infections. However, a smaller percentage of patients (n=76, 19%) reported that they would be dissatisfied if their healthcare provider did not prescribe antibiotics for a respiratory tract infection. Only 48 (12%) patients expected their healthcare provider to prescribe antibiotics if they asked for them (Figure 2).

Factors influencing antibiotic use

The results of the logistic regression analyses revealed that patients' expectations for antibiotic prescribing (OR = 2.5, 95% CI = 1.8-3.6) were strongly associated with inappropriate antibiotic use. Patients who expected their healthcare providers to prescribe antibiotics were more likely to report having used antibiotics in the past year. Conversely, patient satisfaction with antibiotic prescribing (OR = 0.6, 95% CI = 0.4-0.9) was negatively associated with inappropriate antibiotic use. Patients who were satisfied with their healthcare provider's decision not to prescribe antibiotics were less likely to report having used antibiotics in the past year. Additionally, health literacy levels (OR = 1.8, 95% CI = 1.2-2.7) were found to be associated with inappropriate antibiotic use, with patients having lower health literacy levels being more likely to report having used antibiotics in the past year.

## Discussion

The results of this study provide important insights into patient attitudes and beliefs about antibiotic use in primary care. Patients had moderate knowledge about antibiotics, with most recognizing the potential side effects and risks associated with their use. However, patients also held several misconceptions about antibiotics, including their effectiveness against viruses and the need for antibiotics for every infection. These findings are consistent with previous research that has shown a lack of knowledge and misunderstandings about antibiotics among the general public [9].

The misuse of antibiotics was significantly linked to patient demands for antibiotic prescriptions. Those patients who held the belief that antibiotics should be prescribed for all types of infections and those who expected their healthcare provider to prescribe antibiotics for respiratory tract infections were more likely to have used antibiotics within the previous year [10]. This discovery highlights the critical role of patient education and communication initiatives targeted at enhancing patient expectations and comprehension of the appropriate use of antibiotics in primary care. Such initiatives could comprise educational resources outlining the disparities between bacterial and viral infections, along with the potential hazards associated with the overuse of antibiotics [10].

It is noteworthy that patient satisfaction regarding antibiotic prescription had an adverse association with the misuse of antibiotics. Patients who expressed satisfaction with their healthcare provider's decision to refrain from prescribing antibiotics were less likely to report antibiotic usage in the past year. This observation indicates that approaches to antibiotic prescribing that prioritize patient preferences and involve shared decision-making between patients and healthcare providers could be efficacious in decreasing superfluous antibiotic use. Prior investigations have demonstrated that patient-centered antibiotic prescribing methods can enhance patient satisfaction and lower the rates of antibiotic prescriptions [11-13].

The findings underscore the significance of health literacy in the context of antibiotic use. Individuals with lower educational levels were more inclined to disclose having utilized antibiotics within the preceding year. This finding aligns with earlier studies that have linked health literacy to medication usage [14,15]. Interventions aimed at improving health literacy may be effective in reducing inappropriate antibiotic use among patients with lower health literacy levels.

The study has some constraints worth noting. Firstly, the survey used self-reported information, which may be affected by recall bias and social desirability bias. Secondly, the sample was limited to patients from primary care clinics in Jeddah only, which might not accurately represent other patient groups. Future investigations should endeavor to confirm these results in larger and more diverse patient cohorts. Additionally, longitudinal studies could be carried out to analyze the evolution of patient attitudes and convictions regarding the use of antibiotics over time.

## Conclusions

This study highlights the importance of patient education and communication interventions in promoting appropriate antibiotic use in primary care. Patients' attitudes and beliefs, including their expectations for antibiotic prescribing and their health literacy levels, were found to be strong predictors of inappropriate antibiotic use. Overall, the study emphasizes the need for a collaborative effort between healthcare providers and patients to promote the appropriate use of antibiotics and preserve the effectiveness of these important medications.
